# Endocannabinoids, Anandamide and 2-Arachidonoylglycerol, as Prognostic Markers of Sepsis Outcome and Complications

**DOI:** 10.1089/can.2022.0046

**Published:** 2023-10-09

**Authors:** Ines Šahinović, Sanja Mandić, Damir Mihić, Mario Duvnjak, Domagoj Loinjak, Dario Sabadi, Zlatko Majić, Ljiljana Perić, Vatroslav Šerić

**Affiliations:** ^1^ Department of Clinical Laboratory Diagnostics, University Hospital Osijek, Osijek, Croatia.; ^2^ J.J. Strossmayer University of Osijek, Faculty of Medicine Osijek, Osijek, Croatia.; ^3^ Department of Pulmonology and Intensive Care, Clinic of Internal Medicine, University Hospital Osijek, Osijek, Croatia.; ^4^ Clinic of Infective Diseases, University Hospital Osijek, Osijek, Croatia.

**Keywords:** 2-arachidonoylglycerol, anandamide, endocannabinoids, invasive mechanical ventilation, prognosis, sepsis

## Abstract

**Background:**

One of the major challenges in improving sepsis care is early prediction of sepsis complications. The endocannabinoid system has been intensely studied in recent years; however, little is known about its role in sepsis in humans. This study aimed to assess the prognostic role of endocannabinoids, anandamide (AEA) and 2-arachidonoylglycerol (2-AG), as early predictors of mortality, invasive mechanical ventilation (IMV) requirement, and length of stay (LOS) in patients with sepsis.

**Materials and Methods:**

In total, 106 patients with confirmed sepsis were enrolled in this study. The patients were divided into groups according to mortality outcome (survival, *N*=53; nonsurvival, *N*=53), IMV requirement (IMV group, *N*=26; non-IMV group, *N*=80), and LOS (LOS <10 days, *N*=59; LOS ≥10 days, *N*=47). Patients' clinical status was assessed along with laboratory biomarkers as well as AEA and 2-AG concentration measurements early on admission to emergency units. AEA and 2-AG levels were measured by enzyme-linked immunosorbent assay (ELISA) using an ELISA processor, EtiMax 3000 (DiaSorin, Saluggia, Italy). The predictive value of AEA and 2-AG for the studied sepsis outcomes and complications was analyzed using univariate and multivariate analyses and receiver operating characteristic (ROC) curve analysis.

**Results:**

Two endocannabinoids showed no significant difference between survivors and nonsurvivors, although an AEA concentration <7.16 μg/L predicted mortality outcome with a sensitivity of 57% (95% confidence interval [CI] 42–71) and specificity of 80% (95% CI 66–91). AEA concentrations ≤17.84 μg/L predicted LOS ≥10 days with sensitivity of 98% (95% CI 89–100) and specificity of 34% (95% CI 22–47). When analyzing IMV requirement, levels of AEA and 2-AG were significantly lower within the IMV group compared with the non-IMV group (5.94 μg/L [2.04–9.44] and 6.70 μg/L [3.50–27.04], *p*=0.043, and 5.68 μg/L [2.30–8.60] and 9.58 μg/L [4.83–40.05], *p*=0.002, respectively). The 2-AG showed the best performance for IMV requirement prediction, with both sensitivity and specificity of 69% (*p*<0.001). Endocannabinoid AEA was an independent risk factor of LOS ≥10 days (odds ratio [OR] 23.59; 95% CI 3.03–183.83; *p*=0.003) and IMV requirement in sepsis (OR 0.79; 95% CI, 0.67–0.93; *p*=0.004).

**Conclusion:**

Low AEA concentration is a prognostic factor of hospital LOS longer than 10 days. Lower AEA and 2-AG concentrations obtained at the time of admission to the hospital are predictors of IMV requirement.

## Introduction

Sepsis is a complex, heterogeneous syndrome that causes multiple organ dysfunction due to a dysregulated host response to infection.^1^ Despite a better understanding of its pathophysiology and improved management, sepsis remains a major clinical challenge due to its fearsome complications. The most prevalent complications of sepsis are acute respiratory and cardiovascular failure, requiring intensive care unit admission and, in some cases, invasive mechanical ventilation (IMV) initiation.^2^ The mortality rate associated with sepsis complications remains extremely high. Approximately 20% of the all-cause deaths worldwide are caused by sepsis.^3^

It is important to identify early predictors of sepsis complications and mortality so that time-correct and adequate interventions can be planned to prevent unwanted outcomes.

The endocannabinoid system is an evolutionarily conserved system that has been widely studied over the past two decades and is recognized as a key regulator of immune functions in inflammation. It consists of three main elements that mediate its functions and signaling: bioactive lipid mediators—endocannabinoids, endocannabinoid receptors, and proteins and enzymes that mediate the synthesis, transport, and degradation of endocannabinoids.^4–7^ Two main endocannabinoids are arachidonoylethanolamide (anandamide [AEA]) and 2-arachidonoylglycerol (2-AG).

The importance of endocannabinoids in immune system regulation has been confirmed in sepsis studies using animal models. Endocannabinoids have shown a series of anti-inflammatory,^8,9^ antioxidant,^10,11^ proapoptotic,^12^ and immunoregulatory^13^ properties in sepsis. Activation of endocannabinoid receptors on immune cells results in a decrease in proinflammatory cytokine release^14,15^ and reduces the neutrophil and macrophage recruitment.^16^ Multicellular damage caused by proinflammatory and pro-oxidative mediators leads to multiple organ dysfunction, worsening of the patient's general medical condition, and finally, death. Considering the role of endocannabinoids, we hypothesized that they could be early predictors of severe events in sepsis.

The aim of our study was to assess the prognostic value of endocannabinoids, AEA and 2-AG, as early predictors of mortality, IMV requirement, and length of stay (LOS) in sepsis.

### Subjects and Methods

#### Study design

A prospective observational cohort study was conducted at the University Hospital Osijek, Croatia, over a 15-month period (February 2018 to April 2019). The study was approved by the Ethics Committee of the University Hospital Osijek and was completed in accordance with the Declaration of Helsinki.

#### Subjects

Patients over 18 years of age, admitted with suspected sepsis to the Emergency Unit of the Department of Infectious Disease and the Department of Emergency Medicine and then hospitalized at the Department of Infectious Diseases or the Intensive Care Unit, were considered for the study. Suspected sepsis was defined as a set of clinical symptoms followed by the results of radiological, laboratory, and microbiological analyses. Organ dysfunction was assessed using the Sepsis-related Organ Failure Assessment (SOFA) score. Patients were treated according to the institutional protocol for sepsis management based on recommendations from the Surviving Sepsis Campaign.^17^ Only patients with a clinically and laboratory-confirmed sepsis diagnosis were eligible for the study. All sepsis subjects were followed up until lethal outcome or hospital discharge.

Patients were excluded from the study if they were <18 years old, did not have a confirmed sepsis diagnosis, were transferred to another health institution, or had recently undergone surgery.

**FIG. 1.**
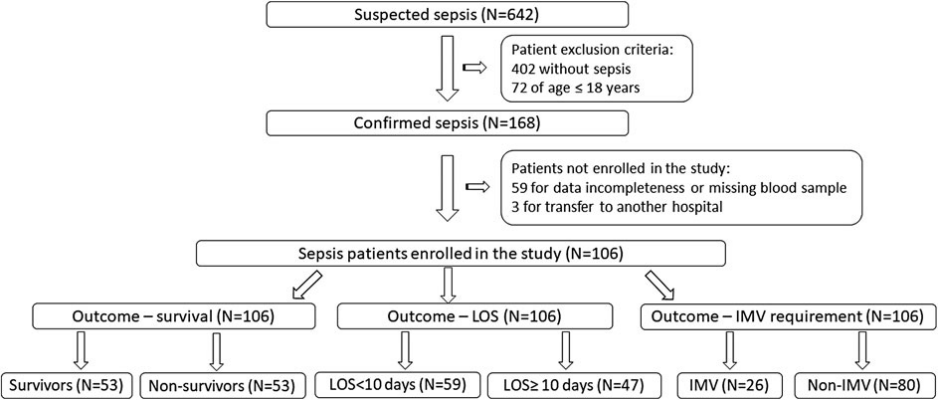
Flowchart of the study population selection for analyzing sepsis outcome: survival, LOS and IMV requirement. IMV, invasive mechanical ventilation; LOS, length of stay.

In total, 106 patients were included in this study. They were classified based on mortality (survivors, *N*=53, and nonsurvivors, *N*=53), LOS outcome (LOS <10 days, *N*=59, and LOS ≥10 days, *N*=47), and developed complications (patients who required IMV, *N*=26, and patients with no need for IMV, *N*=80). Patient enrollment in the study and their classifications are shown in Figure 1.

Routine blood analysis was performed immediately after admission to the emergency units. Samples were obtained by venipuncture before antibiotic administration. Blood samples were collected in 3-mL EDTA tube, 2.7-mL sodium citrate tube, and 6-mL serum-separating tube (BD Vacutainer, Becton, Dickinson, and Company, Franklin Lakes, New York, USA). The samples were centrifuged at 1370 *g* for 10 min. Aliquots of EDTA plasma and serum were separated within 2 h of sampling and frozen at −20°C until analysis of endocannabinoids and interleukin (IL)-6, but not longer than 7 months. Culture analysis of various body fluids (blood, urine, abscess, and catheter swabs) was performed in 92 patients.

## Materials and Methods

Age, sex, blood pressure, oxygen saturation of arterial blood, body fluid culture results, and infection site were documented from the electronic medical records of the hospital informatics system. Mean arterial pressure was calculated as 1/3 (systolic blood pressure) +2/3 (diastolic blood pressure).

Basic hematology (complete blood count), coagulation (prothrombin time, fibrinogen, and D-dimer), and biochemistry analyses (urea, creatinine, C-reactive protein [CRP], and procalcitonin [PCT]) were performed immediately upon admission to emergency units. Complete blood count was performed using a hematology analyzer Sysmex XN-2000 (Sysmex Corporation, Kobe, Japan). Coagulation analyses were performed using a BCS XP coagulometer (Siemens Healthineers AG, Erlangen, Germany). Biochemistry analyses were determined on biochemistry analyzer Olympus AU680 (Beckman Coulter, Brea, CA, USA) using a spectrophotometric assay (urea), an enzymatic assay with creatininase (creatinine), and a latex-enhanced immunoturbidimetric assay (CRP). PCT level was measured using an electrochemiluminescence immunoassay (ECLIA) on an immunochemistry analyzer, Cobas e411 (Roche Diagnostics GmbH, Mannheim, Germany).

Serum IL-6 and plasma endocannabinoids, anandamide and 2-AG levels, were measured using a commercially available Enzyme-Linked Immunosorbent Assay (ELISA). IL-6 levels were measured using sandwich ELISA according to the manufacturer's instructions (Invitrogen; Thermo Fisher Scientific, Waltham, MA, USA). A 10-fold serum sample predilution was made. The endocannabinoids, anandamide and 2-AG, were measured in plasma samples and kept on ice after sample thawing. Sample predilution of 1:4 for anandamide and 1:5 for 2-AG was performed with phosphate-buffered saline, pH 7.2. Endocannabinoids were measured using competitive ELISA according to the manufacturer's instructions (Abbexa, Milton, Cambridge, United Kingdom) on an ELISA processor EtiMax 3000 (DiaSorin, Saluggia, Italy). AEA and 2-AG intra- and interassay variabilities were <10% and <12%, respectively. Standard curves with the corresponding equations obtained by ELISA are provided in the Supplementary Figures 1 and 2.

### Statistical analysis

Data distribution was tested using the Shapiro–Wilk test. Results are presented as medians and interquartile ranges. Differences between the groups were estimated using the Mann–Whitney *U* test. The strength and direction of association between data were determined using the Spearman's rank coefficient, *ρ*. The prognostic accuracy of endocannabinoids and inflammatory biomarkers (CRP, PCT, and IL-6) in predicting sepsis outcomes and complications was assessed using receiver operating characteristic (ROC) curve analysis. The best cutoff was calculated using Youden's Index with the highest sum of sensitivity and specificity. Independent risk factors for 28-day mortality were identified using Cox proportional hazard regression analysis. IMV requirement and hospital LOS risk factors were assessed by the logistic regression analysis. Regression analyses of risk factors for the studied outcomes were performed in two steps. In the first step, univariate analyses were performed for demographic and laboratory factors.

All factors with a *p*-value <0.1 in the univariate regression analysis were entered into the stepwise Cox or logistic regression models. Risk factors for mortality outcome, LOS, and IMV requirement were expressed as hazard ratios (HRs) and odds ratios (ORs). Area under the curve (AUC), HR, and OR are presented with 95% confidence intervals. Statistical analysis was performed using the MedCalc Statistical Software Version 12.4.0.0 (MedCalc Software, Mariakerke, Belgium). All testing were two-tailed, and statistical significance was set at *p*<0.05.

## Results

### Baseline clinical and laboratory findings

Several clinical and laboratory markers have been used to assess the outcomes of sepsis. The baseline clinical and laboratory findings are presented in Table 1.

**Table 1.** Baseline Characteristics of the Patients According to the Studied Outcomes

Variable	*Sepsis survivors (*N*=53)*	*Sepsis nonsurvivors (*N*=53)*	*p*	*IMV (*N*=26)*	*Non-IMV (*N*=80)*	*p*
Demographics						
Male, *N* (%)	22 (42)	20 (38)	0.843^*^	10 (39)	32 (40)	0.927^*^
Age, median (range)	71 (27–94)	75 (35–92)	0.117^*^	70 (39–84)	73 (27–94)	0.120^*^
Hospitalization, median (IQR)						
Length of stay (LOS)	9 (8–14)	8 (5–14)	0.071^‡^	13 (7–26)	9 (6–13)	0.036^‡^
Infection site, *N* (%)						
Respiratory tract	18 (34)	25 (47)	0.235^*^	20 (77)	23 (29)	<0.001^*^
Genitourinary tract	21 (40)	18 (34)	0.687^*^	2 (8)	37 (46)	0.003^†^
Other diseases^a^	10 (18)	7 (13)	0.296^†^	3 (12)	14 (18)	0.556^†^
Undefined origin	4 (8)	3 (6)	1.000^†^	1 (3)	6 (3	1.000^†^
Body fluid culture, *N* (%)						
Total 92 (87)						
Positive 52 (57)	23 (44)	29 (56)	0.327^*^	32 (61)	20 (39)	0.031^*^
Sepsis score, median (IQR)						
qSOFA	1 (1–2)	2 (2–3)	<0.001^‡^	2 (2–3)	1 (1–2)	0.002^‡^
SOFA	8 (7–10)	11 (9–13)	<0.001^‡^	12 (11–15)	8 (6–10)	<0.001^‡^
Laboratory findings, median (IQR)						
White blood cells (×10^9^/L)	15.0 (12.7–16.2)	12.5 (10.9–14.0)	0.091^‡^	12.9 (10.0–17.2)	13.6 (10.6–19.5)	0.602^‡^
Hemoglobin (g/L)	120 (109–126)	104 (95–114)	0.057^‡^	94 (87–117)	118 (101–136)	0.003^‡^
MCHC (g/L)	338 (333–339)	327 (325–331)	0.012^‡^	325 (313–329)	337 (325–343)	0.003^‡^
Prothrombin time (ratio)	0.89 (0.82–1.01)	0.65 (0.56–0.80)	0.006^‡^	0.66 (0.50–0.89)	0.84 (0.57–1.04)	0.171^‡^
D-dimer (μg/L)	2391 (1483–3858)	5587 (3139–7636)	0.020^‡^	2137 (1814–5727)	3681 (1444–7623)	0.674^‡^
Fibrinogen (g/L)	7.1 (6.1–7.7)	6.1 (5,0–6.6)	0.007^‡^	6.5 (5.3–8.7)	6.6 (5.2–7.7)	0.720^‡^
CRP (mg/L)	222.1 (176.4–296.8)	253.2 (187.1–312.9)	0.245^‡^	282.9 (229.3–352.0)	217.8 (170.7–295.4)	0.008^‡^
Procalcitonin (μg/L)	4.66 (1.63–18.99)	5.57 (2.38–17.74)	0.870^‡^	5.80 (2.41–21.81)	4.21 (0.84–18.44)	0.249^‡^
IL-6 (ng/L)	60.2 (32.3–179.7)	85.8 (44.8–195.5)	0.134^‡^	93.2 (71.9–227.1)	64.3 (33.7–181.4)	0.073^‡^
Urea (mmol/L)	10.6 (8.5–15.0)	20.9 (16.2–27.1)	<0.001^‡^	22.6 (12.4–31.4)	14.0 (8.9–21.2)	0.032^‡^
Creatinine (μmol/L)	136 (113–152)	168 (148–250)	0.020^‡^	282 (108–423)	142 (97–215)	0.025^‡^
Endocannabinoids, median (IQR)						
Anandamide (μg/L)	5.04 (3.74–6.75)	8.40 (5.01–22.25)	0.057^‡^	5.94 (2.04–9.44)	6.70 (3.50–27.04)	0.043^‡^
2-arachidonoylglycerol (μg/L)	7.25 (4.28–15.95)	8.35 (3.28–29.34)	0.980^‡^	5.68 (2.30–8.60)	9.58 (4.83–40.05)	0.002^‡^

^a^ Other diseases: malignancy, infection of central venous catheter, gastroenterocolitis, ileus, acute cholecystitis.

CRP, C-reactive protein; IMV, invasive mechanical ventilation; IQR, interquartile range; IL, interleukin; LOS, length of stay; MCHC, mean cell hemoglobin concentration; qSOFA, quick Sequential Organ Failure Assessment; SOFA, Sequential Organ Failure Assessment.

Statistics: ^*^Chi-square test; ^†^Fisher's exact test; ^‡^Mann–Whitney test.

No differences in age or sex were observed between groups. The most prevalent underlying causes of sepsis were pneumonia and urinary tract infection, which together represented 79% of the sepsis cohort. Seven patients had sepsis of undefined origin. Pneumonia was the predominant cause of sepsis in the IMV group (77%). Body fluid cultures were obtained from 92 patients (87%), of whom 52 (57%) were culture positive. Among the identified microbes, *Escherichia coli* was the most commonly isolated pathogen (32%), followed by *Streptococcus pneumoniae* (18%) and methicillin-resistant *Staphylococcus aureus* (18%).

Kidney dysfunction was significantly associated with mortality and IMV requirement outcomes. There were no significant differences in inflammatory biomarkers (white blood cells [WBC], CRP, PCT, and IL-6) between survivors and nonsurvivors at admission. However, the CRP levels were significantly higher in the IMV group compared with the non-IMV group.

**FIG. 2.**
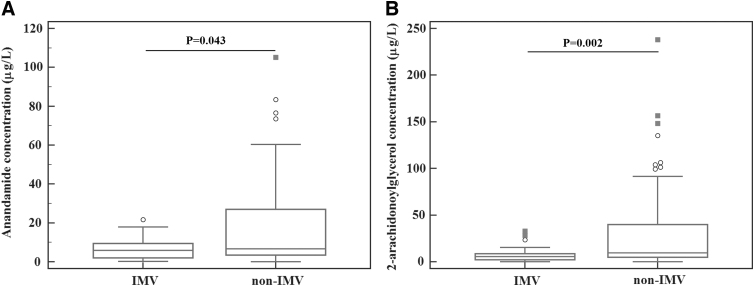
Anandamide **(A)** and 2-AG **(B)** at admission in septic patients requiring IMV versus patients nonrequiring IMV (non-IMV). 2-AG, 2-arachidonoylglycerol; non-IMV, nonrequiring invasive mechanical ventilation.

We observed no significant differences in endocannabinoid concentrations between survivors and nonsurvivors. When analyzing IMV requirement outcome, the levels of AEA and 2-AG were lower in the IMV group compared with the non-IMV group of patients (Fig. 2). Furthermore, AEA concentrations showed to be higher in patients hospitalized for less than 10 days compared with those hospitalized for 10 and more days (7.12 μg/L; 3.44–29.90, and 5.68 μg/L; 3.25–9.18, *p*=0.028, respectively). The 2-AG showed no significant difference between the two LOS groups (8.60 μg/L; 4.4–45.0, and 7.20 μg/L; 3.88–13.73 μg/L, *p*=0.352, respectively).

We observed a significant correlation between AEA and 2-AG in the three studied sepsis outcomes, indicating that endocannabinoids tended to increase together in sepsis (Table 2).

**Table 2.** Correlation of Endocannabinoids Anandamide and 2-Arachidonoylglycerol in Sepsis

AEA and 2-AG	Spearman's R (95% CI)	*p* ^*^
Survivors	0.82 (0.70–0.89)	<0.001
Nonsurvivors	0.78 (0.65–0.87)	<0.001
IMV group	0.56 (0.22–0.78)	0.003
Non-IMV group	0.85 (0.77–0.90)	<0.001
LOS <10 days	0.81 (0.71–0.89)	<0.001
LOS ≥10 days	0.60 (0.37–0.75)	<0.001

^*^ *p* Value is two-tailed.

2-AG, 2-arachidonoylglycerol; AEA, anandamide; CI, confidence interval.

### Prediction of mortality and IMV requirement

ROC curves were constructed for endocannabinoids and inflammatory biomarkers (CRP, PCT, and IL-6) to identify cutoff values that predict outcomes at admission. Only AEA at the admission point showed significance in mortality prediction with an AUC of 0.67 (95% CI 0.57–0.76; *p*=0.003). The optimal cutoff value, calculated using Youden's Index, was >7.16 μg/L, with a sensitivity of 57% (95% CI 42–71) and a specificity of 80% (95% CI 66–91). Positive and negative likelihood ratios (LR+ and LR−) were 2.9 (95% CI 1.5–5.5) and 0.5 (95% CI 0.4–0.8), respectively.

**FIG. 3.**
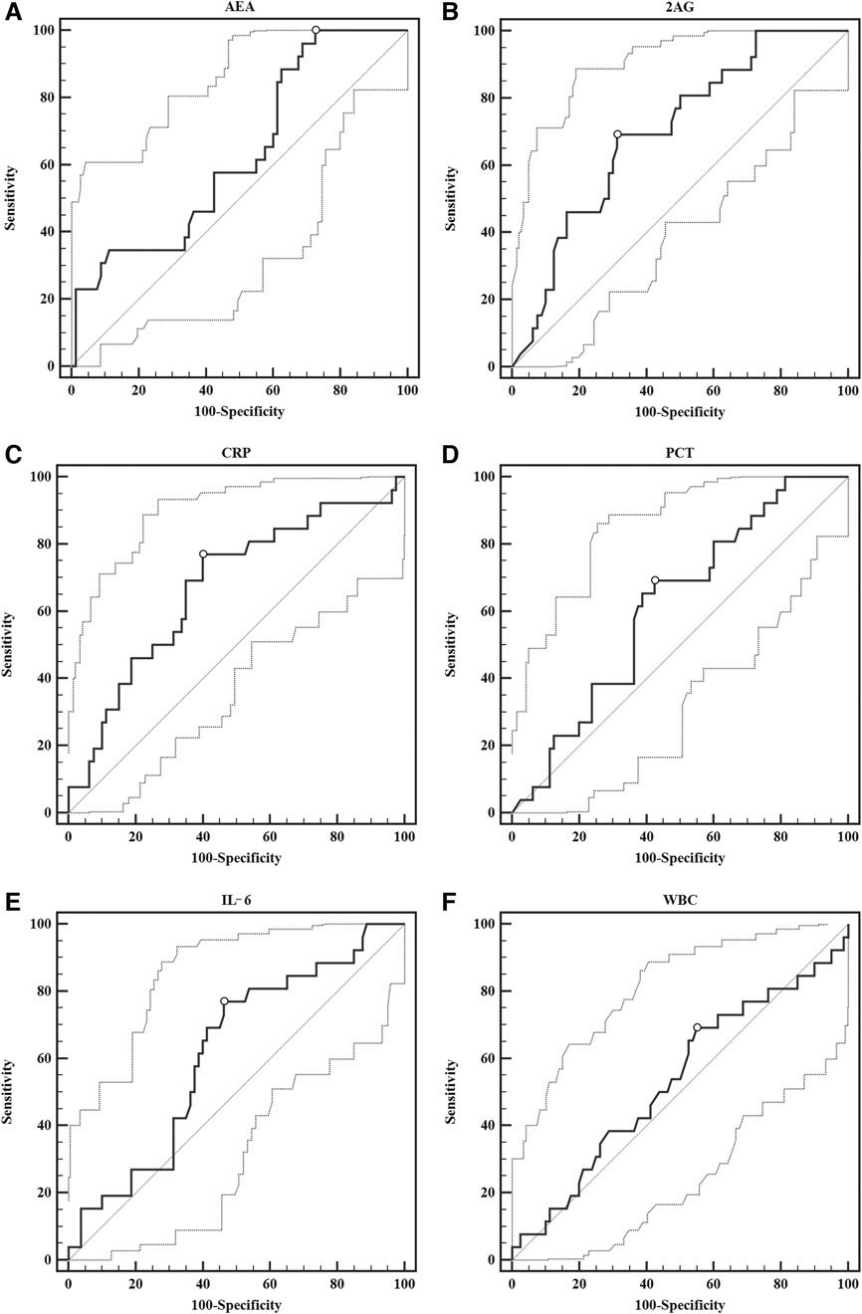
ROC of endocannabinoids and inflammatory biomarkers in prediction of IMV requirement in sepsis. **(A)** AEA; **(B)** 2-AG; **(C)** CRP; **(D)** PCT; **(E)** IL-6; **(F)** WBC. AEA, anandamide; CRP, C-reactive protein; IL-6, interleukin-6; PCT, procalcitonin; ROC, receiver operating curve; WBC, white blood cells.

For IMV requirement prediction, AEA at a cutoff value of ≤21.64 μg/L showed the highest sensitivity (100%) compared with other biomarkers, but poor specificity (28%). The 2-AG showed the best performance for IMV requirement prognosis with both sensitivity and specificity of 69%, and the highest LR+ of 2.2 among the analyzed biomarkers (Table 3 and Fig. 3).

**Table 3.** Receiver Operating Curve Analysis of Endocannabinoids and Inflammatory Biomarkers in Prediction of Invasive Mechanical Ventilation Requirement and Length of Hospital Stay Greater Than 10 Days in Sepsis

Variable	AUC (95% CI)	*p*	Cutoff	Sensitivity, % (95% CI)	Specificity, % (95% CI)	LR+ (95% CI)	LR− (95% CI)
Invasive mechanical ventilation							
AEA (μg/L)	0.63 (0.53–0.72)	0.032	≤21.64	100 (87–100)	28 (18–39)	1.4 (1.2–1.6)	0
2-AG (μg/L)	0.70 (0.60–0.79)	<0.001	≤6.60	69 (48–86)	69 (57–79)	2.2 (1.5–3.4)	0.5 (0.2–0.8)
CRP (mg/L)	0.67 (0.58–0.76)	0.005	>227.6	77 (56–91)	60 (48–71)	1.9 (1.4–2.7)	0.4 (0.2–0.8)
PCT (μg/L)	0.62 (0.52–0.71)	0.057	>4.21	69 (48–86)	58 (46–69)	1.6 (1.1–2.3)	0.5 (0.3–1.0)
IL-6 (ng/L)	0.62 (0.52–0.71)	0.053	>69.1	77 (56–91)	54 (42–65)	1.7 (1.2–2.3)	0.4 (0.2–0.9)
WBC (×10^9^/L)	0.53 (0.44–0.63)	0.610	≤14.9	69 (48–86)	45 (34–57)	1.3 (0.9–1.7)	0.7 (0.4–1.3)
Length of stay for 10 or more days (LOS ≥10)							
AEA (μg/L)	0.63 (0.53–0.72)	0.021	≤17.84	98 (89–100)	34 (22–47)	1.48	0.06
2-AG (μg/L)	0.55 (0.45–0.65)	0.350	≤15.40	79 (64–89)	39 (27–53)	1.29	0.55
CRP (mg/L)	0.59 (0.49–0.69)	0.094	>187.1	85 (72–94)	39 (27–53)	1.39	0.38
PCT (μg/L)	0.54 (0.40–0.67)	0.641	≥48.6	97 (82–100)	24 (10–44)	1.27	0.14
IL-6 (ng/L)	0.51 (0.41–0.61)	0.872	>154.9	17 (8–31)	63 (49–75)	0.46	1.32
WBC (×10^9^/L)	0.57 (0.47–0.67)	0.188	>11.3	72 (57–84)	44 (31–58)	1.29	0.63

AUC, area under the curve; LR, likelihood ratio; PCT, procalcitonin; WBC, white blood cells.

In the evaluation of endocannabinoids and inflammatory biomarkers' prediction of LOS outcome, AEA showed to be the only significant prognostic factor. Concentrations ≤17.84 μg/L predicted LOS ≥10 days with high sensitivity of 98% (95% CI 89–100) (Table 3).

### Prognostic value of biomarkers

Cox proportional hazard regression analysis of 28-day mortality showed that endocannabinoids, AEA and 2-AG, were not mortality risk factors in patients with sepsis. The significant risk factors determined by multivariate analysis were fibrinogen (HR 0.79; 95% CI 0.64–0.97; *p*=0.026), WBC count (HR 0.90; 95% CI 0.83–0.99; *p*=0.028), mean corpuscular hemoglobin concentration (HR 0.95; 95% CI 0.92–0.99; *p*=0.008), and SOFA score (HR 1.17; 95% CI 1.03–1.33; *p*=0.018).

Endocannabinoid AEA was found to be an independent risk factor for IMV requirement and LOS in sepsis. At admission, a lower plasma AEA concentration was associated with a higher risk of IMV requirement (Table 4) and longer hospital LOS in patients with sepsis (OR 23.59; 95% CI 3.03–183.83, *p*=0.003).

**Table 4.** Univariate and Multivariate Logistic Regression Analysis of Risk Factors for Invasive Mechanical Ventilation Requirement in Sepsis

	Univariate logistic regression analysis		Multivariate logistic regression analysis	
Variable	OR (95%CI)	*p*	OR (95%CI)	*p*
Age	0.97 (0.93–0.99)	0.026		
qSOFA	2.35 (1.35–4.07)	0.002	0.12 (0.02–0.69)	0.018
SOFA	1.83 (1.43–2.35)	<0.001	4.57 (1.87–11.18)	<0.001
MAP	0.95 (0.92–0.98)	0.002		
Body weight	0.97 (0.94–0.99)	0.036		
O_2_ saturation (art)	0.92 (0.88–0.96)	<0.001	0.79 (0.67–0.93)	0.005
AEA	0.93 (0.88–0.99)	0.027	0.79 (0.67–0.93)	0.004
2-AG	0.95 (0.91–0.99)	0.035		
CRP	1.01 (1.00–1.01)	0.017		
PCT	0.99 (0.99–1.01)	0.659		
IL-6	1.00 (0.99–1.01)	0.152		
Hemoglobin	0.97 (0.95–0.99)	0.005		
MCHC	0.95 (0.92–0.99)	0.003		
Fibrinogen	1.07 (0.84–1.36)	0.568		
D-dimer	1.00 (0.99–1.00)	0.226		
Urea	1.04 (0.99–1.08)	0.055		
Creatinine	1.00 (1.00–1.01)	0.006		

MAP, mean arterial pressure; O_2_ saturation (art), oxygen saturation of arterial blood; OR, odds ratio.

## Discussion

This study evaluated the prognostic value of endocannabinoids, AEA and 2-AG, in patients with sepsis at the time of a hospital admission.

Our results showed that lower baseline plasma AEA and 2-AG concentrations were prognostic factors for IMV requirement in patients with sepsis. Furthermore, AEA concentration was shown to be a predictor of hospital LOS longer than 10 days. This finding indicates a possible protective role of the endocannabinoid AEA against more severe inflammation and its consumption in sepsis with multiple complications. According to our study, AEA is also an independent risk factor for IMV requirements, with high sensitivity at a cutoff value of 21.64 μg/L. This finding is consistent with research done by Ladak who also reported lower AEA concentrations in septic patients requiring IMV. They measured a median AEA concentration of 0.20 μg/L in mechanically ventilated patients and 0.35 μg/L in nonventilated patients (*p*=0.008).^18^

There is growing evidence of an endocannabinoid role in sepsis and acute respiratory distress syndrome (ARDS) caused by inflammation. Most of this knowledge comes from animal models of sepsis. Inhibition of endocannabinoid-degrading enzymes (fatty acid amide hydrolase and monoacylglycerol lipase [MAGL]) attenuated ARDS in animal models by increasing the concentrations of AEA and 2-AG.^19,20^

The increase in endocannabinoids within the lungs prevented airway hyperreactivity and inflammation.^21,22^ AEA reduced proinflammatory cytokines (IL-2, tumor necrosis factor-alpha, interferon-gamma) and immune cells (T-lymphocytes and neutrophils) within the lungs while increasing the action of anti-inflammatory immune cells such as T-regulatory lymphocytes. The mechanism of this anti-inflammatory and immunosuppressive action that leads to the attenuation of ARDS in mice is the regulation and control of proinflammatory gene expression.^23^ Our results are consistent with previous studies showing that lower baseline AEA and 2-AG concentrations are associated with greater inflammation and organ dysfunction. Alveolar macrophages (AMs) represent the first line of defense within the lungs. They express cannabinoid receptors and can synthesize AEA and 2-AG.^24,25^ The 2-AG is metabolized in AMs by cyclooxygenase-2 into prostaglandin D2 glycerol and prostaglandin E2 glycerol, which reduces lipopolysaccharide (LPS)-induced lung inflammation in mice.^26^

We also found a strong correlation between the two endocannabinoids, indicating that they tend to increase together during sepsis. The exceptions were the IMV and LOS ≥10 days' groups, which showed a weaker AEA and 2-AG correlation. Most septic patients with a LOS ≥10 days required IMV and had worse clinical outcomes and more sepsis complications. Although the levels of both endocannabinoids were significantly lower in the IMV group, we observed a larger decrease in 2-AG than in AEA concentration. The difference between 2-AG and AEA in the IMV group was 0.37 (−3.51 to 4.54), and in the non-IMV group 3.81 (0.92–14.94). The reasons for these observations are not fully understood. Some *in vitro* studies have shown a protective role of 2-AG within the lungs. Costola-de-Souza et al. showed that the inhibition of MAGL, an enzyme that hydrolyzes 2-AG and terminates it's signaling, attenuates airway inflammation in a murine model of LPS-induced ARDS.

Mice with increased 2-AG within the lungs showed decreased WBC migration to the lungs, decreased vascular permeability, airway hyperreactivity, and cytokine concentrations.^19^ A study by Weis et al. showed that the 2-AG concentration negatively correlates with IMV duration in patients undergoing cardiac surgery with cardiopulmonary bypass (*ρ*=−0.43, *p*=0.03).^27^ The 2-AG might be an important anti-inflammatory and protective factor in ARDS, but further research of this potential role in humans is needed.

Only a few studies have assessed the role of endocannabinoids in sepsis in humans, and the results are contradictory. Kohro et al. studied the role of polymyxin B-hemofiltration on the AEA concentration in 24 septic patients and showed higher AEA levels in nonsurvivors.^28^ Ladak studied AEA, entourage lipids oleoylethanolamide, and palmitoylethanolamide in 49 septic patients at admission point to hospital and showed no difference between sepsis survivors and nonsurvivors.^18^ Our study showed that endocannabinoids are not sepsis mortality risk factors.

The current understanding of sepsis immunopathogenesis involves a biphasic pathology. The early phase of sepsis is characterized by a hyperinflammatory response. If there is no balance between pro- and anti-inflammatory mechanisms, sepsis advances into a second, hypoinflammatory phase.^29^ The subjects evaluated at the hospital admission were in the early hyperinflammatory phase of sepsis development with increased proinflammatory biomarkers (Table 1). Our study results indicate that the endocannabinoid system tends to be activated early at the beginning of the hyperinflammatory phase of sepsis. AEA and 2-AG concentrations were lower in subjects with more severe damage and respiratory system function impairment, which might indicate an anti-inflammatory role of endocannabinoids.

The limitation of this study was the variable time of patient presentation from the onset of sepsis symptoms. Some patients presented with mild and some with severe symptoms of inflammation. The nonsurvivors and the IMV group presented to the hospital with more advanced systemic inflammation. We consider that this is often a clinical reality in emergency departments. In this study, we aimed to study the role of endocannabinoids as risk factors in everyday clinical practice. To date, only a few studies dealing with the role of AEA and 2-AG in patients with sepsis have been published. To our knowledge, only one such study evaluating the role of AEA in sepsis has been conducted to date, but on a much smaller number of septic patients (*N*=49).^18^ There is still a need for further research, especially in lethal outcome risk assessment.

One of the limitations of this study was the exclusion of patients with a previous surgery. Patients with postoperative sepsis are admitted for treatment long before the onset of symptoms. There may be a significant influence of therapy received before and during surgery. Schelling et al. studied the effect of general anesthesia on blood endocannabinoids levels. They observed a marked decrease in AEA concentration after etomidate/sevoflurane administration.^30^ Furthermore, some studies have shown that surgical procedures have a significant effect on endocannabinoid levels.^27^ Further studies are required to evaluate the role of endocannabinoids in the development of postoperative sepsis.

To the best of our knowledge, this is the first study to report the prognostic role of endocannabinoids, AEA and 2-AG, as independent risk factors for outcomes such as mortality, LOS, and IMV requirement in sepsis. Our study on the role of endocannabinoids as early risk factors of sepsis outcome and complications resulted in the first report of AEA and 2-AG as predictors of IMV requirement and AEA as predictor of LOS even early on hospital admission. These findings encourage further studies on the role of the endocannabinoid system in sepsis and airway function in humans. IMV is nowadays a very current topic due to its requirement in severe cases of COVID-19 disease.^31–33^ Multiple articles have shown the potential of AEA to decrease levels of IL-6, a main IL in the cytokine storm.^34–36^

In conclusion, the results of our study support the involvement of AEA and 2-AG in sepsis. The baseline AEA and 2-AG concentrations predict IMV requirement early at hospital admission. AEA has also shown to be a predictor of hospital LOS. The ability to early identify patients at higher risk for sepsis complications may encourage timely intervention and result in better clinical outcomes.

## Supplementary Material

Supplemental data

Supplemental data

## Abbreviations Used

2-AG: 2-arachidonoylglycerol
AEA: anandamide
AM: alveolar macrophage
ARDS: acute respiratory distress syndrome
AUC: area under the curve
CI: confidence interval
CRP: C-reactive protein
ELISA: Enzyme-Linked Immunosorbent Assay
HR: hazard ratio
IL: interleukin
IMV: invasive mechanical ventilation
LOS: length of stay
LPS: lipopolysaccharide
LR−: negative likelihood ratio
LR+: positive likelihood ratio
MAGL: monoacylglycerol lipase
MAP: mean arterial pressure
MCHC: mean cell hemoglobin concentration
OR: odds ratio
PCT: procalcitonin
ROC: receiver operating characteristic
SOFA: Sepsis-related Organ Failure Assessment
WBC: white blood cells
