# Supplementary figures and images for: Endocannabinoids, Anandamide and 2-Arachidonoylglycerol, as Prognostic Markers of Sepsis Outcome and Complications
Source: Cannabis Cannabinoid Res. 2023 Oct 9;8(5):802–11. doi: 10.1089/can.2022.0046 (PMC10589499; doi:10.1089/can.2022.0046)

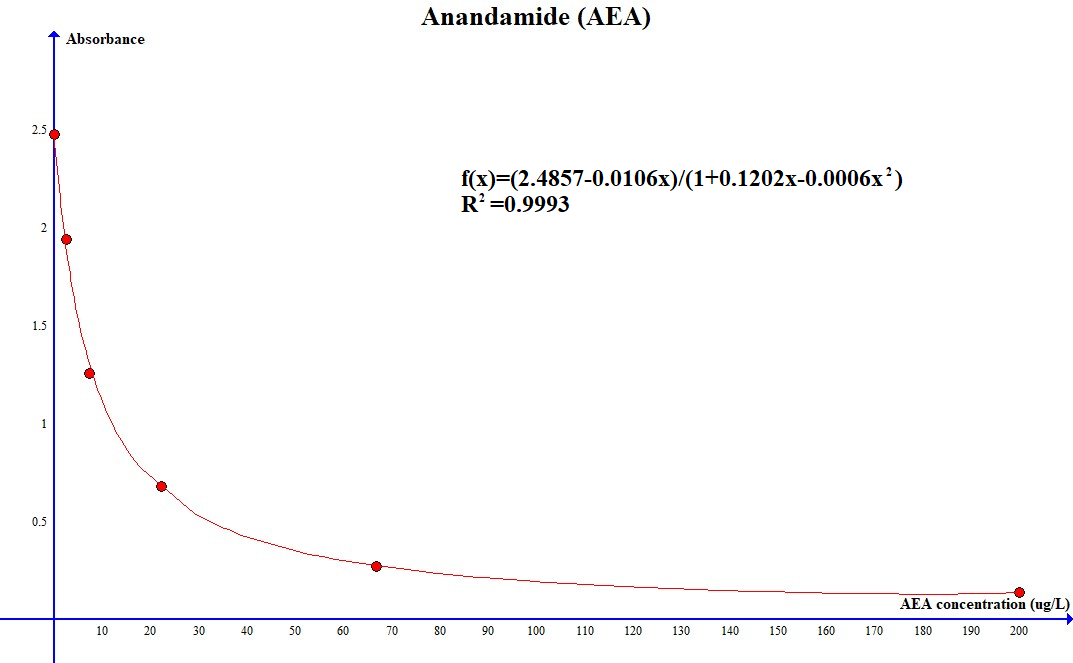

Supplement: Supplemental data [file Supp_FigS1.docx]

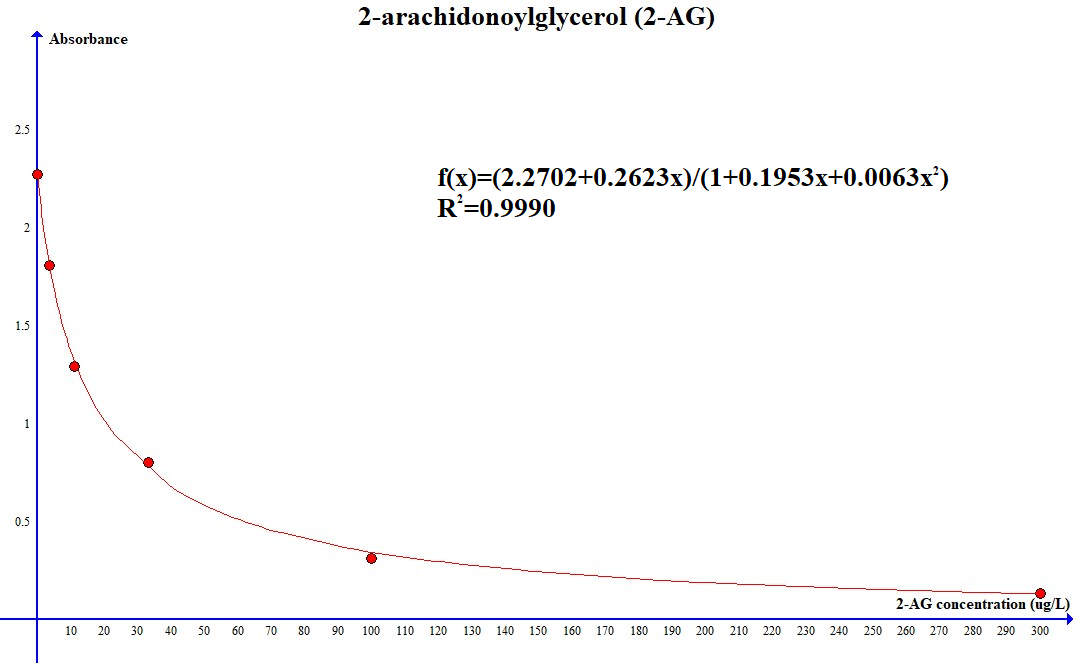

Supplement: Supplemental data [file Supp_FigS2.docx]
